# Supplementary material for: Response to self-care practice messages among patients with diabetes mellitus visiting Jimma University medical center facility based cross sectional design application of extended parallel process model
Source: PLoS One. 2021 Dec 31;16(12):e0261836. doi: 10.1371/journal.pone.0261836 (PMC8719745; doi:10.1371/journal.pone.0261836)
Supplement: S1 File — (DOCX) [file pone.0261836.s001.docx]

## **Annex 1 English Questionnaire**

Direction *1:* Now you are expected to fill about your socio-demographic characteristics. Please
answer by circling your choice and fill in the blank spaces for others.

| S.no | Question/variable | Response |
| --- | --- | --- |
| SD 001 | Age of the respondent | ______ years |
| SD 002 | Sex of the respondent | 1. Male 2. Female |
| SD 003 | What is your marital status? | 1.Single  2.Married  3.Divorced  4.Widowed |
| SD 004 | Religion of respondent? | 1. Muslim  2. Orthodox  3. Protestant  4. Catholic  5.Other specify______ |
| SD 005 | What is your ethnicity? | 1. Oromo  2. Amhara  3. Gurage  4. Kaffa  5. Dawuro  6. Other specify_______ |
| SD 006 | What is your level of education? | 1.Can’t read and write  2. grade completed________  3 Other |
| SD 007 | Monthly income | ____________ |
| SD 008 | Distance to the nearest health facility in km | ____________ |
| SD 009 | What is your occupation | 1.Farmer  2.Merchant  3.Housewife,  4.Government employee  5.Other Specify_______ |
| SD 010 | How long have you been since diagnosed with diabetes mellitus | _________ |
| SD 011 | How long have you been on treatment | __________ |
| SD 012 | Which diabetic patient type | 1. Type 1 2. Type 2 |

**Part 2: Perceived threat of Diabetes complication among patients with diabetes**

Direction 2: now I am going to ask you some questions about your beliefs about the likelihood of experiencing harmful consequences from diabetes. [Read the responses, & check ‘√” infront of each question under the responded option]

SD=Strongly Disagree

D= Disagree

Und=Undecided

A=Agree

SA=Strongly Agree

| **No** | **Questions** | **Response category** | | |  |  |
| --- | --- | --- | --- | --- | --- | --- |
|  |  | **SD** | **D** | **und** | **A** | **SA** |
|  |  | 1 | 2 | 3 | 4 | 5 |
| **Perceived susceptibility to diabetes complication** | | | | | | |
| PT 001 | As **patients with diabetes** , I am at risk of getting diseases like (kidney , heart and hypertension |  |  |  |  |  |
| PT 002 | As **patients with diabetes** , it is possible through process that I will get diseases like  (kidney, heart, hypertension) |  |  |  |  |  |
| PT 003 | As **patients with diabetes** , I have a chance of getting foot ulcer/gangrene |  |  |  |  |  |
| PT 004 | As **patients with diabetes** , I have a chance of experiencing hypoglycemia |  |  |  |  |  |
| **Perceived severity of diabetes complication** | | | | | | |
| PT 005 | Experiencing diseases like kidney, heart and hypertension is a serious problem to **patients with diabetes** |  |  |  |  |  |
| PT 006 | Getting diseases like kidney, heart and hypertension is life threating to **patients with diabetes** |  |  |  |  |  |
| PT 007 | Getting foot ulcer/gangrene leads **patients with diabetes** to loss of body parts. |  |  |  |  |  |
| PT 008 | Experiencing hypoglycemia can lead **patients with diabetes** to sudden deaths |  |  |  |  |  |

**Part 3: Perceived Efficacy of the recommended self-care practice among patients with diabetes**

Direction 3 :Now I am going to ask you some questions about your beliefs on your ability, easiness and effectiveness of self-care practice in deterring diabetes complication. [Read the responses, & check ‘√” in front of each question under the responded option]

| **No** | Questions | **Response category** | | |  |  |
| --- | --- | --- | --- | --- | --- | --- |
|  |  | **SD** | **D** | **N** | **A** | **SA** |
|  |  | **1** | **2** | **3** | **4** | **5** |
| **Perceived Response efficacy of self-care practice** | | | | | | |
| PE001 | For **patients with diabetes** , engaging on regular physical exercise prevents from risks of diseases like kidney, heart and hypertension |  |  |  |  |  |
| PE002 | For **patients with diabetes** , consuming foods like vegetables, fruits, low salt etc] preventsrisks from diseases like kidney, heart and hypertension |  |  |  |  |  |
| PE003 | For **patients with diabetes** , regularly checkup my blood glucose prevents sudden death from. hypoglycemia? |  |  |  |  |  |
| PE004 | For **patients with diabetes** , caring for foot prevents loss of body parts from gangrene |  |  |  |  |  |
| **Perceived Self-efficacy to perform self-care practice** | | | | | | |
| PE 005 | As **patients with diabetes** , it is easy for me to engage on regular physical exercise prevent risks from diseases like kidney, heart and hypertension |  |  |  |  |  |
| PE 006 | As **patients with diabetes** , I am able to adapt consuming foods [like vegetables, fruits, low salt etc.] to prevent risks from diseases like kidney, heart and hypertension |  |  |  |  |  |
| PE 007 | As **patients with diabetes** , I am confident to regularly checkup my blood glucose to prevent hypoglycemia |  |  |  |  |  |
| PE 008 | As **patients with diabetes** , it easy for me to care for my foot to prevent loss of body parts from gangrene |  |  |  |  |  |

**Part 4: Cues to action related to diabetes mellitus**

Direction 4: Now I am going to ask you some questions aboutthings that triggers/motivates you to do self-care practice. [Read the responses, &check ‘√” in front of each question under the responded option]

| **No** | Questions | 1. Yes | 2. No |
| --- | --- | --- | --- |
| C 001 | Do you have a family member with diabetes complication? |  |  |
| C 002 | Have you ever seen /heard about a person who follow recommended self-care practice in last one month |  |  |
| C 003 | Have you ever seen /heard of person having diabetes complication in the last one month |  |  |
| C 004 | Have you ever heard through media about recommended self-care practice during last one month? |  |  |

**Part 5: Diabetes knowledge assessment among patients with diabetes**

Direction 5: Now I am going to ask you some questions about your knowledge regarding diabetes and its complication. [Read the responses, & check circle in front of each question under the responded option]

| **No** | Questions | Response category | | Skip |
| --- | --- | --- | --- | --- |
| K 001 | Diabetes is chronic (lifelong) disease | 1 yes | 0 No |  |
| K 002 | diabetes is curable | 1. Yes | 0. No |  |
| K 004 | Tick that are ways of controlling (managing)diabetes  **Instruction: [don’t read option, give one more chance to mention any thing remaining after ticking all responses given** diabetes | 1. Diet only 2. Regular Exercise 3. Measuring of blood glucose 4. Taking drug 5. I don’t know 6. Other (specify) | |  |
| K 007 | Tick that are the signs of diabetes mellitus  **Instruction: [don’t read option, give one more chance to mention any thing remaining after ticking all responses given** | 1. Polyphagia  2. Polydipsia.  3. Polyuria.  4. Weakness.  5.i don’t know  6 Specify if any_______ | |  |
| K 009 | Tick that are complication of diabetes mellitus?  **Instruction: [don’t read option, give one more chance to mention any thing remaining after ticking all responses given** | 1 Hypoglycemia  2 Foot ulcer/ Gangrene  3 Nerve problems  4 Eye problems  5 Heart problem  6 Kidney problems  7 Hypertension  8 I don’t know  9 Other specify_____ | |  |

**Part 6: Message exposure and recall to diabetes self-care among diabetes patient**

Direction 6: Now I am going to ask you some questions about your Message exposure and recall to diabetes self-care. [Read the responses, & circle in front of each question under the responded option]

| M 001 | Have you heard about diabetes self-care in the last 6 month | yes  No |
| --- | --- | --- |
| M 002 | From where you received information about diabetes self-care practice  **Instruction: [don’t read option, give one more chance to mention any thing remaining after ticking all responses given]** | 1. Health institutions 2. Religious institutions 3. Friends 4. Parents/Spouse 5. Television 6. Radio 7. posters 8. leaflets /brochures 9. Others |
| M 003 | Preferred channels to hear/see about diabetes self-care practice **Instruction: [don’t read option, give one more chance to mention any thing remaining after ticking all responses given]** | 1. Television 2. Radio 3. Peer discussions 4. posters 5. Leaflets/brochures 6. other |
| M 004 | Frequently heardMessage/behavior about diabetes self-care practice  **Instruction: [don’t read option, give one more chance to mention any thing remaining after ticking all responses given]** | 1. Dietary practice 2. Regular physical exercise 3. Foot care 4. Self-blood glucose monitoring |
| M 006 | Preferred messageAppeals of diabetic self-care practice | 1. Dramatic/funny 2. Fear arousal messages |

**Part 7: Diabetes self-care activities during the past 7 days.**

Direction 7: Now I am going to ask you some questions about yourself-care practice of the past seven days. [Read the responses, &check “√” in front of each question under the responded option]

| No | Questions | Response | | | | | | | |
| --- | --- | --- | --- | --- | --- | --- | --- | --- | --- |
|  |  | 0 | 1 | 2 | 3 | 4 | 5 | 6 | 7 |
| SC 001 | How many of the last SEVEN DAYS have you followed a healthful eating plan? |  |  |  |  |  |  |  |  |
| SC 002 | On average, over the past month, how many DAYS PER WEEK have you followed your eating plan? |  |  |  |  |  |  |  |  |
| SC 003 | On how many of the last SEVEN DAYS did you three servings of fruits and vegetables? |  |  |  |  |  |  |  |  |
| SC 004 | On how many of the last SEVEN DAYS did you eat high fat foods such as red meat ? |  |  |  |  |  |  |  |  |
| SC 005 | On how many of the last SEVEN DAYS did you participate in at least 30 minutes of physical activity? (Total minutes of continuous activity, including walking) |  |  |  |  |  |  |  |  |
| SC 006 | On how many of the last SEVEN DAYS did you participate in a specific exercise session (such as swimming, walking, biking) other than what you do around the house or as part of your work? |  |  |  |  |  |  |  |  |
| SC 007 | On how many of the last SEVEN DAYS didyou test your blood sugar? |  |  |  |  |  |  |  |  |
| SC 008 | On how many of the last SEVEN DAYS didyou test your blood sugar the number oftimes recommended by your health care provider? |  |  |  |  |  |  |  |  |
| SC 009 | On how many of the last SEVEN DAYS didyou check your feet? |  |  |  |  |  |  |  |  |
| SC 010 | On how many of the last SEVEN DAYS didyou inspect the inside of your shoes? |  |  |  |  |  |  |  |  |

**Part 8: Defensive avoidance of patients with diabetes-to-diabetes complication**

Direction 8: Now I am going to ask you some questions about your thought about diabetes complication. [Read the responses, &check “√” in front of each question under the responded option]

| No | Questionnaire | **Response categories** | | | | |
| --- | --- | --- | --- | --- | --- | --- |
|  |  | **SD** | **D** | **N** | **A** | **SA** |
|  |  | **1** | **2** | **3** | **4** | **5** |
| D 001 | I didn’t want to think about my risk for Diabetes complication |  |  |  |  |  |
| D 002 | I didn’t want to do anything to prevent diabetes complication |  |  |  |  |  |
| D 003 | I didn’t want to protect myself from diabetes complication |  |  |  |  |  |
| D 004 | I didn’t want to think about it at all |  |  |  |  |  |
