# Supplementary material for: Response to self-care practice messages among patients with diabetes mellitus visiting Jimma University medical center facility based cross sectional design application of extended parallel process model
Source: PLoS One. 2021 Dec 31;16(12):e0261836. doi: 10.1371/journal.pone.0261836 (PMC8719745; doi:10.1371/journal.pone.0261836)
Supplement: Attachment — Submitted filename: response to reviewers.docx [file pone.0261836.s003.docx]

**Reviewer 1**

Dear Reviewer 1 I have read your comments and I find them helpful and make corrections based on your comments.

**Reviewer 2**

Dear Reviewer 2 I have read your comments and I find them helpful and make corrections based on your comments.
