# Supplementary material for: Response to self-care practice messages among patients with diabetes mellitus visiting Jimma University medical center facility based cross sectional design application of extended parallel process model
Source: PLoS One. 2021 Dec 31;16(12):e0261836. doi: 10.1371/journal.pone.0261836 (PMC8719745; doi:10.1371/journal.pone.0261836)
Supplement: Attachment — Submitted filename: Responses to Editor and Reviewers comments.docx [file pone.0261836.s004.docx]

**Responses to Editor and Reviewers’ Comments**

**Full Title:** entitled Response to Self-Care Practice Messages Among Patients with Diabetes Mellitus Visiting Jimma University Medical Center: Application of Extended Parallel Process Model: Facility Based Cross Sectional Study

Manuscript Number: PONE-D-21-17765R1

**Corresponding Author: Mohammed Jemal**

We are very much grateful for the Editor and reviewers’ time and willingness to review the manuscript. The authors found the comments of the reviewers and editor are very important. The authors were really happy to address the comments raised by reviewers and editor for this manuscript. The authors seriously considered these comments and have gone through the entire body of the manuscript to make necessary editorial corrections and clarifications to the concerns. Corrections are shown by highlighting the texts in yellow color in the main document.

**Reviewer(s)' Comments to Author:**

**Reviewer: 1**

**Comments to the Author**

**Comment 1**: I prefer to use patients with diabetes instead of diabetic. It means that we are setting the identity/name on people without asking them if they agree.
**Response:** Thank you for the comments. Now we replaced diabetic patients to patients with diabetes Please check (**Page 1; line 1 ;page 2; line 1 and line 2; Page 4;line 1,line 16,19 and 23;page 10 line 1;page 11 line 7 and 10;page 12 line 1 )**.

**Comment 2:** I cannot find the word danger in description of the variables. I suggest not to use word danger

**Response:** Thank you. We really appreciate the comment. Now we added the word danger control in the variable section but unfortunately it is must to use the word danger in the application of EPPM model as one of the outcome variables is danger control respondents which are respondents that control the danger of diabetes by taking preventive measure.

So the word danger is a technical word in this study Please check (**Page 18; line 18)**.

**Comment 3:** Please send me the PDF you have in the end of the paper, I could not open it.

**Response:** Many thanks for the comment. We attached the PDF during the submission.

**Comment 4:** First title in reference list is spelled wrong: America Diabets.

**Response:** We really appreciate the comment. Now the word is corrected as “America Diabetes” **Please check (Page 24; line 1).**

**Reviewer: 2**

**Comments to the Author**

Reviewer #2: Jemal et al report a survey on the perceptions on how patients’ access, process, and respond to diabetic self-care messages in Ethiopia. Although an interesting study, there are several major concerns with objectives, terminologies, methods, interpretation, and presentation of findings.
Major concerns:
**Comment 1:** The study objectives are not clearly stated and some are misleading. For example, the authors list: “To describe perceived threat of diabetes patient” and “ To describe perceived efficacy of diabetes patient”.
It is not clear how the survey is achieving the above when the survey was designed to assess the response to self-care message?
The authors must keep the objectives focused and achievable.

**Response:** Thank you for the comment. Now we have changed those misleading objectives to more clear objectives which is “to assess response to self-care practice message among diabetes patient “Please check (**Page 4; line 1)**.

**Comment 2:** The wordings used are also somewhat incomprehensible and inconsistent. What is meant by “control the danger of diabetes? This is used in the abstract and elsewhere.
Please rephrase as appropriate

**Response:** We really appreciate the comment. To define controlling the danger of diabetes according to Extended Parallel Process Model it is a self-protective motivation i.e. performing diabetes self-care practice under a message's recommendations, since we are applying the EPPM model we cannot rephrase the word as it is one of the constructs of the model. Please check (**Page 18; line 18)**.

**Comment 3:** In the methods, the authors need to specify the measuring method in the paper instead of only explaining the meaning of each measurement.
• How reliable is the “Extended Parallel Process Model”?
• whether a high score is better or a low score is better. Has it been validated?

**Response:** We really appreciate the comment. The tool was adopted from the Risk Behaviour Diagnostic scale which is validated tool when applying EPPM in different contexts Now, we have explained the meaning of each measurements and higher score shows better. The reliability was checked for the major constructs of EPPM and it showed its reliable measurement in the context of this study **Please check** (**Page 6; line 8 ;9;13 and 14; page 8 line 11,12)**.

**Comment 4:** Highlight the limitation of a single centre study and therefore may have limited generalizability even within Ethiopia
**Response:** Many thanks for the comments. Now we have highlighted the limitation of a single centre study
**Please check (Page 2; line 23 ;page 15 line 9).**

**Comment 5:** Sample size: The sample size was estimated based on the proportion of danger control response. But I cannot figure out that ‘danger control response’ is a main outcome for the analysis based on the method.

**Response:** Thank you. When applying Extended Parallel process model the outcome variable is danger control response and fear control response which can be achieved through two ways 1) Using discriminatory value (DV) when using this method individuals who scored above 1 (DV>1) are in danger control response **Please check (Page 6; line 27-29).** and

2) The second method is using efficacy threat interaction in this method respondents who scored above median are in danger control response category. In this case responsive and proactive respondents are danger control respondents according to Extended Parallel Process Model **Please check (Page 7; line 8 and 9; line 12-14).**

**Comment 6:** 1) Results: Page 13, last paragraph: The author reported R=0.487, please explain what analysis method was used to obtain the ‘R’.

**Response:** Thank you for the comments. We used Pearson correlation coefficient method to see the relationship between selfcare message response and self-care practice Please **check (Page 10; line 9-10)**

**Comment 7:** The results in Table 6 are confusing to me. How did the author define the subgroups (proactive, fear control, responsive, and no response) from Efficacy Threat Interaction? I cannot find it in the methods section.
**Response:** Many thank for the comments. Now we improved A median split was performed on both threat and efficacy; in both cases respondents at or below the median were placed into the ‘‘low’’ group, and respondents above the median were placed in the ‘‘high’’ group **Please check (Page 8; line 5-7)**.

Responsive respondents are those who scored above median for both Threat and Efficacy **Please check (Page 7; line 8-9)**.

Proactive respondents are those who score above the median for efficacy but below the median for threat **Please check (Page 7; line 13-15)**.

Fear control respondents are those who scored above the median for threat but below the median for Efficacy (**Please check (Page 7; line 10-12)**.

No response respondents are those who scored below the median for both threat and efficacy **Please check (Page 7; line 16-17)**.

**Comment 8)** Page 16, table 8:
a. How did the author define the binary outcome ‘characterization of perceptions toward diabetes mellitus and selfcare practice among diabetes mellitus’?

**Response: -** Many thank for the comments. Now we have defined the outcome variable in to danger control response and fear control response based on Discriminatory value.

Respondents who scored DV≥1 are danger control response and those who scored DV≤ 0 are fear control response respondents. We also changed the title from “characterization of perceptions toward diabetes mellitus and selfcare practice among diabetes mellitus’ into” Response to Self-Care Practice Messages Among Patients with Diabetes Mellitus Visiting Jimma University Medical Centre: Application of Extended Parallel Process Model: Facility Based Cross Sectional Study “**Please check (Page 23; line 1).**

**Comment 9.**  Did the author only control for the factors reported in table 8 or only retained significant predictors in the model? This needs to be explained in the analysis method section. If the author only controlled for the factors in table 8 in the model, please explain why other factors were not adjusted for. Moreover, the factors in table 8 were not defined in the methods.

**Response: -** Many thank for the comments**.** Variables with p value < 0.25 were selected as a candidate variable for multivariable logistic regression. Those significant variables ( P value <0.05) was retained in the final model. **Please check (Page 8; line 9-11)**.

Moreover, we have added the definition of factors in table 8. **Please check (Page 5; line 14-17)**.

**Comment 10** Please attach the survey questionnaire

**Response: -** Many thank for the comments. The questionnaire is attached as a file named “supporting information”
